# The Current State of Mobile Apps Owned by Large Pediatric Hospitals in the United States: Systematic Search and Analysis on Google Play and Apple App Stores

**DOI:** 10.2196/38940

**Published:** 2022-10-06

**Authors:** Tyler Lieser, Yungui Huang, Emre Sezgin

**Affiliations:** 1 The Abigail Wexner Research Institute Nationwide Children's Hospital Columbus, OH United States

**Keywords:** pediatric, child, hospital, mobile app, mobile health, mHealth, health app, digital health, eHealth, hospital-owned app, telehealth, review, app feature, accessibility, patient experience, functionality

## Abstract

**Background:**

Pediatric hospitals in the United States are increasingly leveraging patient-facing mobile apps as their digital front doors for patients, families, and caretakers. These mobile health apps are sanctioned by pediatric hospitals to inform the public or populations about pediatric care to provide individualized information, to enhance communication, and to improve patient experience. Yet the functionalities and user feedback of these hospital mobile apps have not been systematically investigated.

**Objective:**

Our aim was to understand the current state of hospital-owned mobile apps provided by large pediatric hospitals, comparatively analyze and report the services provided, and identify potential gaps to inform developers and providers. The American Hospital Association defines large hospitals as those having a bed count of more than 400.

**Methods:**

We conducted a systematic search on Google Play and Apple App Store to identify all hospital-owned mobile apps from the large pediatric hospitals included in our review. Our inclusion criteria were (1) apps provided by large pediatric hospitals; (2) hospital-owned apps available in Apple App Store and Google Play; and (3) apps that are provided for general populations. Specialty apps that serve specific user groups or populations focusing on education, telehealth, specific conditions or procedures, or apps intended for research or clinician use were excluded. The features and functionality of the included apps were examined.

**Results:**

Of the 16 pediatric hospitals included in our review, 4 (25%) had no general patient-facing apps, 4 (25%) had one app, and 8 (50%) had more than one app available on Google Play or Apple App Store. The 12 hospitals with at least one mobile app had a combined total of 72 apps. Of these 72 apps, 61 (85%) were considered specialty and were excluded from our review, leaving a total of 11 (15%) apps to analyze. Among the 11 apps analyzed, the most common feature was appointment scheduling or reminder (n=9, 82%). Doctor search (n=8, 73%) and patient resources (n=8, 73%) were the second most common, followed by payment, billing, or claims (n=7, 64%), patient portal integration (n=6, 55%), personal health management (n=6, 55%), hospital way finding (n=5, 45%), message a provider (n=4, 36%), urgent care wait times (n=4, 36%), video chat (n=4, 36%), and health information access (n=4, 36%). Parking information (n=3, 27%) was the least common.

**Conclusions:**

Out of the 16 pediatric hospitals identified for our review, 75% (n=12) offer mobile apps. Based on the most common features, these apps were intended to help improve accessibility for patients and families in terms of finding providers, scheduling appointments, and accessing patient resources. We believe the findings will inform pediatric hospital administrators, developers, and other stakeholders to improve app feature offerings and increase their impact on service accessibility and patient experience.

## Introduction

Ownership of smartphones has continued to increase since their introduction in the mid-2000s. According to Pew Research, 85% of Americans own a smartphone, and over half (53%) of US adults own a tablet computer [1]. The way we interact and engage with the world has become increasingly mobile. Currently, there are over 4.8 million apps available on the Apple App Store and Google Play [2] with over 350,000 of those in the health care domain [3], which shows a more than 3-fold increase since 2014 [4]. This rapid adoption of health care mobile apps shows that more people are using their mobile devices for their health and health care needs. Consumer’s expectations of how they interact with health care organizations are shifting toward a mobile-first mindset.

Similarly, recognizing this trend, hospitals have been offering their own apps to meet this demand. An earlier Accenture report presented that two-thirds of the largest US hospitals offer patient-facing mobile health apps [5]. Yet a number of these apps have been poorly implemented, failing to improve patient engagement or provide services. Out of those hospital-owned apps, few offered expected services or functionalities, and it resulted in 2% of patients using these apps [5]. Similar adoption problems have been observed with mobile patient portals as well [6]. Working with younger patients and parents who are more likely to be digital savvy and have stronger desire to be mobile first, pediatric hospitals have more urgency to adapt to this shifting mindset and needs. Pediatric hospitals need to develop their own mobile apps to improve accessibility to better serve their patients and families. There have been mobile health apps to inform the public or populations about pediatric care [7], yet mobile apps provided by pediatric hospitals have not been widely investigated.

The goal of this study is to investigate the hospital-owned apps by large pediatric hospitals in the United States. Large pediatric hospitals serve a high number of patients with a variety of conditions and different populations. Their web-based presence and supporting tools, built to address the patient’s needs with hospital services and resources, are essential to serve the large patient population and potentially have a larger impact. Large hospitals usually have the financial resources to comprehensively develop their apps, and most of the time, they are the first in the market to provide new health care technologies and solutions. Therefore, this study focuses on the large pediatric hospitals with our aims being (1) to understand the current state of hospital-owned mobile apps provided by large pediatric hospitals, (2) to comparatively analyze and report the services provided, and (3) to identify potential gaps to inform hospital administration as they plan and improve their digital health strategies.

## Methods

### Mobile App Inclusion Criteria

**Figure 1:**
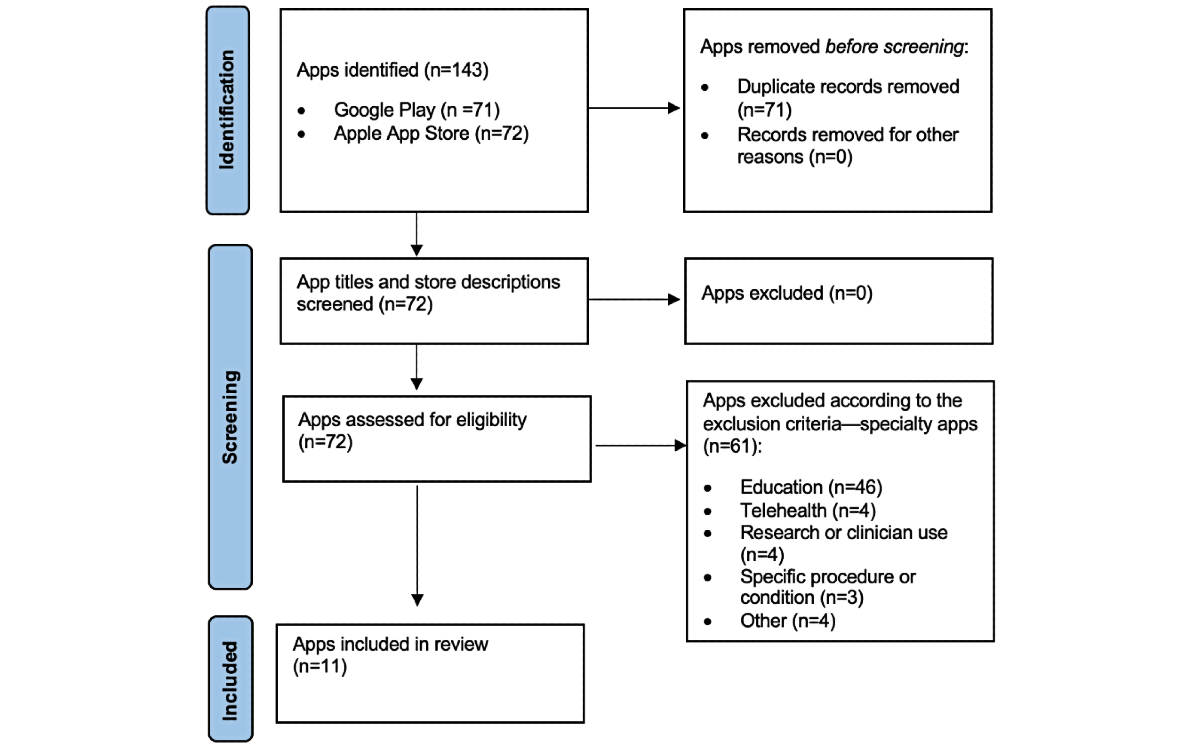
PRISMA (Preferred Reporting Items for Systematic Reviews and Meta-Analyses) flow diagram.

Our inclusion criteria for the health care mobile apps for this study were as follows: (1) they are provided by large pediatric hospitals; (2) they are available in Apple App Store and Google Play; and (3) they are provided for general populations. The American Hospital Association defines large hospitals as having a bed count of more than 400 [8]. Figure 1 shows the PRISMA (Preferred Reporting Items for Systematic Reviews and Meta-Analyses) flow diagram [9], reporting the review procedure.

To identify large pediatric hospitals to include in this review, we leveraged Pediatric Health Information System (PHIS), a comprehensive database with clinical and resource utilization data for inpatient, ambulatory surgery, emergency department, and observation unit encounters for more than 49 children’s hospitals. It is frequently used to study pediatric inpatient care [10]. We queried PHIS, last updated in 2020, to identify pediatric hospitals that had a bed count of 390 or greater, to be inclusive of the hospitals that have a small margin to be rated as large hospitals in the following years.

We used the name of each identified hospital to conduct a systematic search on Google Play and Apple App Store platforms to identify all hospital-owned mobile apps. First, authors identified the keywords. Then, the search was conducted by the first author (TL) between November 2, 2021, and January 14, 2022. Specialty apps centered around education, telehealth, specific conditions or procedures, or apps intended for research or clinician use were excluded, as they are intended to serve a subset of the general population (see Multimedia Appendix 1 for the list of excluded apps). The selected apps were downloaded and reviewed by the authors (TL and ES). We downloaded each app from Google Play on a Google Pixel 4a smartphone to review available features. We used an iPhone 11 to download 1 app that only offered an iOS version (myChop). Out of 16 hospitals, apps were provided by 12 (75%), with a total of 72 hospital-owned and specialty apps. A total of 11 apps met our criteria and were included in this review.

### Mobile App Data Extraction and Analysis

Data available on Google Play and Apple App Store were collected for each app including app name, developer, last update, number of downloads, rating, size, requirements, permissions, as well as app content rating. We used summary statistics to compare features among these hospital mobile apps. To compare features among hospitals, we created a chart and listed the features of each app (Multimedia Appendix 2).

We used appbot.co to conduct sentiment analysis on app reviews. appbot.com was claimed to be trained with 400+ million records and have 93% accuracy [11]. The algorithm analyzes and sorts the reviews into four categories: (1) positive sentiment (accounting in positive comments, eg, “Thanks for this app, it makes life a little more easier”), negative sentiment (accounting in negative comments, eg, “Does not accept same log in as online account...”), neutral sentiment (accounting in comments not having strong sentiment, eg, “I am not sure if I like the new design”), and mixed sentiment (accounting in comments with conflicting sentiment, eg, “Excellent app, with great information, but regrettably have to uninstall due its size”) [12].

## Results

### Overall

A large pediatric hospital identification query to PHIS resulted in 16 pediatric hospitals being included in our review. Of these 16 hospitals, 4 (25%) had no health care mobile apps, 4 (25%) had 1 app, and 8 (50%) had more than one app available on Google Play or Apple App Store. The 12 hospitals with at least one mobile app had a combined total of 72 apps. Of these apps, 61 (85%) were considered specialty and were excluded from our review, leaving a total of 11 (15%) apps for our analysis.

### App Features

A total of 12 features were included in our comparison table (Table 1). The most common feature among apps was appointment scheduling or reminder (n=9, 82%). Doctor search (n=8, 73%) and patient resources (n=8, 73%) were the second most common, followed by payment, billing, or claims (n=7, 64%), patient portal integration (n=6, 55%), personal health management (n=6, 55%), hospital way finding (n=5, 45%), message a provider (n=4, 36%), urgent care wait times (n=4, 36%), video chat (n=4, 36%), and health information access (n=4, 36%). Parking information (n=3, 27%) was the least common.

Appointment scheduling and reminders, the most frequently included feature, allows users to schedule appointments with providers directly within the app. Users will also receive a reminder notification of an approaching appointment. Doctor search gives users the ability to search for providers and review their contact information. Patient resources include features such as FAQs, games, blogs, information about nearby hotels, food, and entertainment. Payment, billing, or claims enable users to see statement balances or claims and make a payment within the app. Patient portal integration allows users to log in to Electronic Health Record patient portal, such as EPIC MyChart, directly from the app. Personal health management features allow users to actively manage their health with features such as requesting prescription refills, listing of medications, dosage and immunizations, as well as the ability to enter and track symptoms and medications. Hospital way finding helps direct users to various locations in the hospital using photos or active navigation. Messaging providers and video chat let users directly message providers and allow them to meet via video. Urgent care wait times allow users to review and receive updates on current wait times at urgent care. Health information access is the feature for the patients and caregivers to access detailed medical information. Finally, parking information lets users see available parking prior to arriving at the hospital.

**Table 1:** Categorized features of the apps.

Features or pediatric hospitals	Texas Children's Hospital	Cincinnati Children's Hospital Medical Center	Nationwide Children's Hospital	Children's Hospital of Philadelphia	Children's Healthcare of Atlanta	Akron Children's Hospital	Children's Health, Dallas	Phoenix Children's	Boston Children's Hospital	St. Louis Children's Hospital	Cook Children's Medical Center	Apps with the listed feature, n (%)
Appointment scheduling and reminder	X^a^	X		X	X	X	X		X	X	X	9 (82)
Doctor search		X		X	X	X	X	X		X	X	8 (73)
Patient resources	X	X	X		X	X	X	X		X		8 (73)
Payment, billing, or claims	X			X		X	X		X	X	X	7 (64)
Patient portal integration			X		X	X	X	X		X		6 (55)
Personal health management		X	X	X	X	X				X		6 (55)
Hospital way finding		X			X	X	X	X				5 (45)
Message provider	X			X					X		X	4 (36)
Urgent care wait times		X	X		X		X					4 (36)
Video chat	X			X			X				X	4 (36)
Health information access				X		X			X		X	4 (36)
Parking information		X					X	X				3 (27)

^a^X: indicates whether or not a hospital's mobile app offers that feature.

### App Data

All apps in this comparison have been updated within the last 2 years, with 82% (9/11) having been updated in 2021. In terms of number of downloads, 55% (6/11) have >10,000 downloads, 27% (3/11) have >5000 downloads, and 1 app has been downloaded >100 times. The number of downloads is only available for Android versions on Google Play store. Therefore, we were unable to find the number of downloads for 1 app, as it is only offered in the iOS version. App size ranged from 16 MB to 152.2 MB with an average size of 68 MB. All apps were available in English with 90% (10/11) also offering one or more additional languages. Texas Children’s, Children’s Healthcare of Atlanta, Akron Children’s, Children’s Health, Phoenix Children’s, St. Louis Children’s, and Cook Children’s Medical Center all offer their app in Spanish, in addition to English. Nationwide Children’s, Children’s Hospital of Philadelphia, Boston Children’s, and Cook Children’s Medical Center offer Spanish and other languages.

### App Ratings and Sentiment Analysis

**Figure 2:**
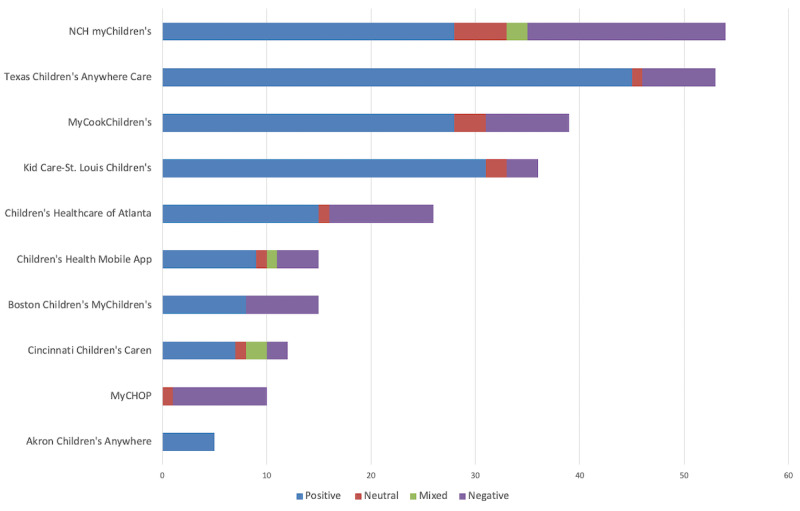
Sentiment analysis distribution (Phoenix Children’s excluded due to 0 reviews). NCH: Nationwide Children's Hospital.

We combined app ratings and number of reviews from both Google Play and Apple App Stores for each app (MyChop was only available in iOS) to determine the average rating and total number of reviews. Ratings for the selected apps, with minimum and maximum allowed as 1 and 5, respectively, ranged from 3.1 to 5, with an average rating of 4.4. Only 265 people left written reviews, and their ratings averaged at 3.4 (Table 2). Texas Children’s Anywhere Care app and Nationwide Children’s myChildren’s app had the highest number of reviews among others (n≥50). The total number of app reviews was 1433. We conducted sentiment analysis (Figure 2) of app reviews using AppBot, a third-party review and ratings analysis tool, to determine positive, neutral, mixed, and negative sentiment of user reviews. Phoenix Children’s was excluded because the app had no reviews.

**Table 2:** App ratings and comments.

Pediatric hospitals	Number of ratings given (n=1433), n (%)	Average rating (out of 5)	Number of app comments (n=265), n (%)	Average rating with comments (out of 5)
Texas Children's Anywhere Care	488 (34)	4.8	53 (20)	4.15
MyCookChildren's	320 (22)	4.7	39 (15)	4.1
NCH^a^ myChildren's	218 (15)	4.2	54 (20)	3.15
Kid Care-St. Louis Children's	105 (7)	4.7	36 (14)	4.55
Children's Healthcare of Atlanta	74 (5)	4.25	26 (10)	3.65
Cincinnati Children's Caren	62 (4)	4.5	12 (5)	4.6
Children's Health Mobile App	61 (4)	4.7	15 (6)	4.05
Boston Children's MyChildren's	40 (3)	3.7	15 (6)	3
MyCHOP	39 (3)	3.1	10 (4)	1.5
Akron Children's Anywhere	20 (1)	4.45	5 (19)	5
Phoenix Children's Hospital	6 (0.04)	5	0 (0)	0

^a^NCH: Nationwide Children's Hospital.

### Mobile Operating System Requirements and Privacy

Software requirements for each app differed, for both Android and iOS platforms. Apps downloaded from Google Play required Android versions ranging from 4.4 (originally released in 2013) to 7 (originally released in 2016). iOS versions of apps were available on iPhone, iPad, iPod Touch, and Mac devices, and required iOS versions 9 (originally released in 2015) to 13.2 (originally released in 2019). In terms of privacy, Texas Children’s Anywhere Care app required the highest number of permissions (24) including access requests to device location, photos, camera, microphone, etc. MyCHOP required the least with no permissions or data being collected from the app.

## Discussion

### Principal Findings

This review provides an overview of current state features and functionalities of large pediatric hospital–owned mobile apps in 2022. Out of 16 large pediatric hospitals in the United States, most of them (n=11, 85%) owned at least one app. This suggests the significance and investments on mobile apps by large pediatric hospitals to support communications with patients and caregivers via smartphones. This finding could be essential for decision makers for pediatric hospital investment strategies in mobile health apps [13]. The 7 hospitals in our review that do not offer any mobile apps may be at risk of seeing a decline in patient satisfaction, as hospital mobile app use has been shown to increase overall patient experience [14]. Hospitals without mobile apps should consider how features made available by other hospital apps could benefit their own patients and families.

Most of the apps offered similar functionalities. We identified personal health management and patient resources, appointment scheduling and reminders, and doctor search as the top features among included apps, followed by payment, billing, or claims, patient portal integration, hospital way finding, message a provider, urgent care wait times, and parking information. These categories suggest that hospitals aim to facilitate primarily remote care and in-hospital navigation and care management over their apps. Such practices can reduce hospitals’ operation costs, improve efficiency [15], and enhance patient engagement [16]. Doctor search as well as appointment scheduling and reminders were top features in a similar app review conducted in Taiwan [17].

### Accessibility

Accessibility is reported in terms of app size, cost, maintenance, and language availability in this section. All the apps have been actively maintained, given the fact that they have been updated within a year period (in 2021 or later). They are all free to download. App sizes ranged from 16 MB to 152.2 MB with an average size of 68 MB. With the minimum storage of modern smartphones at or above 32 GB [18], these free health care mobile apps offer great accessibility in terms of users being able to download and store an app on their phone without sacrificing storage capacity. Nonetheless, there may still be a digital divide such as lack of access to a smartphone, insufficient data plans or internet access, or low digital literacy, which may limit the access of these mobile services for underserved populations (eg, low-income patients, senior citizens, and rural patients). Practitioners should consider the digital divide and barriers in owning and using technologies by the populations; 1 in 5 low-income adults and approximately 30% of senior citizens do not own a smartphone but have a cell phone. In addition, rural residents, racial and ethnic minorities, people living on tribal lands, low-income families, and senior citizens are less likely to have broadband at home [19].

Language could be another potential barrier to patients accessing these apps. Language barriers in health care lead to miscommunication between the medical professional and patient, reducing both parties’ satisfaction and decreasing the quality of health care delivery and patient safety [17,20]. All apps were offered in English, with 90% also offering the app in Spanish. Only 36% (n=11) of hospitals offered their app in more than English and Spanish. Pediatric hospitals serve diverse populations and must account for a broader spectrum of languages.

### App Rating

The total number of ratings among all 11 apps was 1433 with an average rating of 4.4 out of 5. There was a total of 265 app comments among all 11 apps with an average comment rating of 3.4. Overall, the sentiment of comments left by users in the app stores was positive. There was a 1-point rating difference between average ratings among all raters (4.4) and among raters who provided user comments (3.4). This difference may indicate that a higher volume of less satisfied app users are leaving reviews and comments on the app stores versus satisfied users. However, it is hard to quantitatively interpret correlation among user review sentiments and app ratings and review quantity [21].

There is a broad range for the number of ratings per app—Texas Children’s has 488 ratings while Phoenix Children’s has 6. This range may be impacted by factors such as patient volume, location, app functionalities, and more. The apps with high number of ratings and average rating scores and sentiment analysis results could be considered as a benchmark by other hospitals to identify the features to include. For instance, Texas Children’s received the highest average rating and positive sentiment comments (4.8/5). The volume of raters and high ratings may indicate that the features from these apps may offer insight when developing hospital apps (considering potential reviewer biases). Developers should consider user feedback to improve pediatric hospital–owned apps to be more aware of user needs and proactively address app issues and fill the identified gaps [22].

### Privacy

Privacy and security concerns of user data remain one of the top barriers to adoption of mobile health apps. Users could be concerned about what data are being collected and stored, who can access the data, and what purposes the data are being used for [23]. We found that the number of access permissions for pediatric hospital–owned apps goes up to 24 access points, which consist of collecting data from phone sensors and controlling phones (see Multimedia Appendix 1 for permission requests by each app). The MyCHOP app does not collect any data from the user, while Texas Children’s Anywhere Care app requested 24 permissions for data collection. Most of the data collected from apps included location, access to photos, videos and camera, microphone, Wi-Fi connections, and more. Several apps collected data that would not be linked to the user’s identity but may be used for the developer’s advertising or marketing purposes.

Even though privacy is one of the user's concerns, literature shows that most mobile apps do not prioritize privacy of user data. For example, of the 79 mobile health apps certified as being clinically safe and trustworthy by the United Kingdom National Health Service, 89% were found to transfer information online, 66% of which was not encrypted [24]. Developers of pediatric hospital–owned apps must follow privacy policies of hospitals and health institutions to ensure the apps are compliant and collect only necessary data and explain how those data will be used and protected to the end users.

### Limitations

One limitation was the study being limited to large pediatric hospitals in the United States. This limited the study to opt out smaller sized pediatric hospitals or adult hospitals in the United States or abroad. Second, hospitals included in this review were based on bed count. This limitation excludes hospitals that may have lower bed count, but higher number of annual visits. Third, features outlined in this review are subject to change, as these apps are continually being updated on a regular basis. Fourth, we were not able to assess the quality of the mobile apps due to limited access to the apps (without being a hospital patient or having an account). Fifth, due to time and resource constraints, we were not able to analyze user comments to identify which features users felt needed to be improved or which features were lacking from each app. Sixth, the comments and ratings could be impacted from behavioral biases; based on the experience of the reviewer, there could be polarized reviews that we were not able to analyze and identify [25]. In addition, negativity bias or confirmation bias could be considered while reviewing the results [26,27]. Lastly, we did not focus on the impact of these apps to improve patient care or health outcomes. In that regard, future works are suggested to investigate how hospital-owned mobile apps impact patient experience, health outcomes, as well as comparing app quality across hospitals (eg, using mobile app rating scales [28,29]).

Specialty apps (n=61) were excluded from our study, which were provided by specific clinical departments, or focused on research studies, education, telehealth, procedures, or conditions. The number of specialty apps may indicate that app development within some pediatric hospitals is conducted in silos within clinical departments or research groups, which raises questions about their governance, cross-integration, and contributions to the hospital operations. Further studies are suggested toward the specialty apps.

### Conclusions

In this study, we reviewed hospital-owned mobile apps provided by large pediatric hospitals in the United States. Out of 16 hospitals identified, 75% of pediatric hospitals in our review offer mobile apps. Based on the most common features, these apps were intended to help improve accessibility for patients and families in terms of finding providers, scheduling appointments, and accessing patient resources. Inferring actual usage of the health care apps from the number of downloads and user ratings, the adoption of mobile apps is still a major issue. Future works should study the processes that hospitals use when developing mobile apps to ensure user feedback is considered, as well as accessibility and privacy considerations, when determining the features to be implemented. Gathering user feedback will help developers determine the most desired features and may help increase adoption. Developing apps using user-centric, iterative approaches, soliciting inputs from representative user bases, and incorporating feedback from active users will be key to continuously improving health care mobile apps to reach the goals of having them serve as the digital front door, enhancing patient communication and improving patient experience, among others. We believe our findings will inform hospital administrators, developers, practitioners, and other stakeholders to identify and improve app features and services in pediatric hospitals.

## Appendix

Multimedia Appendix 1 This file is a list of apps that met our exclusion criteria and were not included our review.

Multimedia Appendix 2 Spreadsheet containing full app comparison.

## Abbreviations

PHIS: Pediatric Health Information System
PRISMA: Preferred Reporting Items for Systematic Reviews and Meta-Analyses
