# Supplementary material for: The Current State of Mobile Apps Owned by Large Pediatric Hospitals in the United States: Systematic Search and Analysis on Google Play and Apple App Stores
Source: JMIR Pediatr Parent. 2022 Oct 6;5(4):e38940. doi: 10.2196/38940 (PMC9585439; doi:10.2196/38940)
Supplement: Multimedia Appendix 1 [file pediatrics_v5i4e38940_app1.pdf]

Hospital name	App name	Developer	Last updated	Summary	Exclusion criteria		
**Riley Hospital for Children at Indiana University Health**	IU Health Virtual Visits: Online Doctor Visit	Indiana University Health	November 16, 2021	Anyone in Indiana (ages 2+) can now see a highly skilled Indiana University Health provider 24 hours per day, 7 days a week through an online live video with the IU Health Virtual Visits app<br>Virtual Visits can be used for, but not limited to, the following symptoms:<br>• Bronchitis, cold, cough, congestion, fever, flu and other sinus symptoms<br>• Diarrhea, heartburn, nausea and vomiting<br>• Cuts, scrapes, sprains, bruises, burns and insect bites<br>• Hives, pink eye and rash<br>• Infections, including bladder, yeast or urinary tract infection<br><br>We also treat conditions such as acne, allergies, asthma, athlete's foot and back pain.<br>If you use Apple Health, you can choose to share your health information with the provider during your virtual video visit, such as heart rate, blood pressure, body temperature, blood glucose level, weight, nutritional information, and respiratory rate.	Telehealth		
**Children's Hospital of Philadelphia**	Vaccines on the Go	CHOP Applications	May 18, 2021	Vaccines on the Go: What You Should Know contains information about:<br>• Vaccines and the diseases they prevent<br>• Recommended immunization schedules for children, teens and adults<br>• Vaccine safety concerns, including autism, thimerosal and too many vaccines<br>• How the immune system works, including what happens during an infection or when a person is immune compromised<br>• Disease and vaccine information related to travel, including descriptions of cholera, Japanese encephalitis virus, typhoid and yellow fever	Education		
**Children's Hospital of Philadelphia**	iManage SCD	CHOP Applications	June 25, 2021	Learn more about SCD, potential issues and triggers, management techniques, and how you can stay healthy, happy, and live your best life! Join the community and compete with others as you track pain, mood, fitness, and hydration. Learn from your peers, and encourage each other to gain more knowledge and skills as you transition into adult care.	Specific procedure/condition		
**Children's Hospital of Philadelphia**	MyHeart	CHOP Applications	February 14, 2021	This app is for use by invitation only for select patients and research participants at the Children's Hospital of Philadelphia. When used in conjunction with a paired wearable device, this app will allow the CHOP team to help track parameters such as exercise, heart rate, physical activity, nutrition, and any side effects experienced throughout your participation in certain CHOP-based programs and research studies.	Research/clinician use		
**Children's Hospital of Philadelphia**	CHOP Movies	CHOP Applications	June 9, 2021	CHOP Movies-on-Demand offers Children's Hospital of Philadelphia Health patients and families a free library of streaming entertainment featuring a curated selection of comedy, action, drama, and kid-friendly movies.	Other		
**Children's Hospital of Philadelphia**	ADAPTS	CHOP Applications	April 5, 2021	ADAPTS is an app for adolescents and young adults to report on their daily symptoms, mood, stressors, and environment. When users complete surveys, they receive rewards including money, funny memes, and a growing virtual environment with animals and plants.	Other		
**Akron Children's Hospital**	Quick Care	Akron Children's Hospital	July 8, 2021	Quick Care Online connects patients with a board-certified pediatrician, adult physician or advanced practice provider for minor medical conditions through video on a compatible mobile phone, tablet or computer.<br><br>The Quick Care Online app allows subscribers to:<br><br>• Authenticate your Akron Children's Quick Care Online account.<br>• Talk to a board-certified health care provider from anywhere.<br>• Create and update your medical history.<br>• Update your personal, contact, login and billing information.	Telehealth		
**Children's Health, Dallas**	Children's Health VirtualVisit - Family Care 24/7	Children's Health	May 21, 2021	Key features of Virtual Visit include:<br>• Online consultations for children and adults with trusted providers<br>• Affordable visits – $55<br>• Private, secure and HIPPA compliant<br>• Board-certified physicians trained in the U.S.<br>• 24/7 availability – even on holidays<br>• Convenient prescriptions with pick up at your preferred pharmacy	Telehealth		
**Children's Health, Dallas**	Asthma Buddy	Children's Health	September 18, 2020	Children's Health Asthma Buddy provides children with asthma and their parents with a unique set of resources and tools to effectively and proactively manage their asthma.<br>Provides quick access to valuable asthma education and management tools<br>• Helps users track their asthma medication use and set reminders<br>• Allows users to share their Asthma Action Plan and Asthma Control Test (ACT) scores<br>• Encourages users to self-manage their asthma symptoms over time and track improvement<br>• Now includes account creation capabilities that enable you to track your data with across multiple devices	Education		
**Boston Children's Hospital**	Boston Children's Ignyte	Boston Children's Hospital	September 26, 2019	Ignyte is a secure app created by Boston Children's Hospital Cardiac Fitness Program for patients to track physical activity, get motivated, improve confidence, and attain fitness goals.<br>With Ignyte, doctors, trainers, and patients have all the tools at their fingertips to easily and securely track physical activity progress, get motivated, and develop confidence in their ability to get fit. There is a bank of suggested pre-planned workouts as well as the ability for users to customize their own. Positive mindset practices are encouraged and count toward fitness goals to create both a strong body and a strong mind.	Other		
**Boston Children's Hospital**	Boston Children's Scope 360	Boston Children's Hospital	March 18, 2020	Here's how Scope 360° is going to make staying informed easier for you:<br>• Get the latest Boston Children's, industry and local news<br>• Customize your notifications about breaking news and announcements that apply to you!<br>• Connect with the Boston Children's community by posting your own photos, stories and feedback<br>• Quickly get all the news you want so you can get back to the other important matters in your day!	Other		
**Boston Children's Hospital**	Boston Children's HMP	Boston Children's Hospital	February 1, 2019	With the Heart Center Home Monitoring Program, Boston Children's Hospital Heart Center patients can send clinicians key clinical information, including weight, oxygen saturation, formula/fluid amounts and other important information that can help improve your care.	Research/clinician use		
**Boston Children's Hospital**	MyWellNet at Boston Children's	Boston Children's Hospital	April 13, 2017	MyWellNet – designed and developed by a nurse practitioner, this innovative app is used by patients with MS to track and log symptoms and medications, and to communicate real-time with your healthcare provider.<br><br>With MyWellNet you can:<br><br>- Capture and track your MS symptoms with our intuitive graphical interface<br>- Easily log and chart your MS medications<br>- Receive reminders to log your medication<br>- Have real-time communication with your healthcare provider to confirm that your treatment plan is working effectively for you	Specific procedure/condition		
**Boston Children's Hospital**	GI Motility at BCH	Boston Children's Hospital	October 18, 2018	The Center for Motility and Functional GI Disorders at Boston Children's Hospital uses the GIM app as part of clinical care to track patient symptoms, progress, and outcomes.<br><br>Track patients' data such as a patients' gastrointestinal history, symptoms, medications, procedures, allergies, diet changes	Specific procedure/condition		
**Nationwide Children's Hospital**	Make Safe Happen	NCH	December 10, 2019	The Make Safe Happen™ App helps make your home safer for kids with room-by-room advice customized for the ages of the children in the home. Simply select your children's ages, the rooms of your home, and begin making your home safer today.<br><br>• Keep track of your safety progress with an easy checklist design. From anchoring flat-screen TVs to keeping cosmetics out of reach, this home safety app has you covered.<br>• Practice a home fire drill with your family to make sure you all know what to do when the smoke alarms go off.<br>• Need to purchase a safety product? Tap to add it to a shopping list or buy it immediately from Amazon.<br>• Set-up safety reminders to your calendar, like when to change your smoke alarm batteries.<br>• Get safety notifications to help you stay on top of home safety issues as the seasons change and your children grow.<br>• Feel confident knowing that the safety information in this app comes from injury prevention experts at Nationwide Children's Hospital.	Education		
**Nationwide Children's Hospital**	My Residency	NCH	June 2, 2020	"My Residency" – A mobile tool and guide for Resident's at Nationwide Children's Hospital. My Residency is available by invitation only to those Pediatric Resident's at Nationwide Children's Hospital. This helping tool is designed to provide quick access to information vital to the program. Review scheduled lecture times and log your attendance. Review clinical guidelines and reference books. Access your online schedule and online calculators. The App provides access to our phone numbers and connectivity to departments across the system, information on the various rotations, and a directory of other residents.	Research/clinician use		
**Nationwide Children's Hospital**	Physician Connect	NCH	January 14, 2020	With Physician Connect you can access expert pediatric content direct from Nationwide Children's including:<br>• Access free CME On Demand, CME opportunities and Medical Grand Rounds<br>• View the latest protocols and guidelines to help identify, diagnose and manage pediatric conditions<br>• Quick access to Hypertension calculator, Bili Tool, BMI Calculator, STI guidelines, LactMed and MedLine Plus<br>• Make a Referral right from the home screen<br>• Message directly- and securely - with our specialists (if you have an NPI#)<br>• Search best practice Q&A database on a variety of topics<br>• New secure notepad area to keep important info handy and private<br>• Specialized content for your practice such as Behavioral Health, Heart, Gastroenterology, • Pulmonary, Surgical Services and Education (more coming)<br>• Connect directly to Carelink<br>• Quick links to our clinics, programs, locations and services<br>• Hear the latest news and research on PediaCast, our educational podcast for parents<br>• Up-to-date research and news from our parent-education blog, 700Children's	Telehealth		
**Nationwide Children's Hospital**	myNeuroHandbook	NCH	May 14, 2020	"myNeuroHandbook" – A mobile tool and guide for Child Neurology fellows at Nationwide Children's Hospital. My Neuro Handbook is available by invitation only to those Pediatric Residents at Nationwide Children's Hospital. This tool is designed to provide quick access to information vital to patient care and the program. Review online schedule, orientation materials, scheduled lecture times, clinical protocols, medication guidelines, and online references. The App provides access to phone numbers and connectivity to departments across the system.	Research/clinician use		
**Nationwide Children's Hospital**	VARIAT Sim	NCH	October 8, 2020	VARIAT incorporates contextual, experiential learning in a gamified environment to improve awareness of implicit biases among Medicaid providers, educate on how biases and social determinants of health may lead to inequitable care, and offer strategies and resources to minimize health disparities. The game consists of two distinct modules that focus on implicit biases toward sexual orientation and gender identity, race, and socio-economic status within a medical setting.	Education		
Phoenix Children's Hospital	Our Journey with Asthma	Phoenix Children's	February 18, 2020	The Our Journey™ with Asthma app is designed to:<br>— Be used from the time of admission throughout hospitalization, to facilitate conversations<br>between families and the health care team.<br>— Help parents identify what they need to know before going home.<br>— Give parents a safe way to admit what they don't know or understand, and ask questions.<br>— Cue families on what they need to teach back to or demonstrate for their nurse or doctor.<br>This gives the health care provider an opportunity to confirm information and skills and correct misunderstandings.<br>— Help families feel confident that they can safely care for their child at home after discharge<br>from the hospital, know the signs of possible problems, and know how to respond.	Education		
Phoenix Children's Hospital	Nuestro Camino y el asma	Phoenix Children's	February 20, 2020	Spanish version of "Our Journey with Asthma"	Education		
Phoenix Children's Hospital	Our Journey with Autism	Phoenix Children's	February 18, 2020	The Our Journey™ with Autism app is designed to:<br>- Be used from the time of diagnosis throughout the child's journey with autism, to facilitate conversations between families and the health care team.<br>- Help parents identify what they need to know once their child is diagnosed.<br>- Give parents a safe way to admit what they don't know or understand, and ask questions.<br>- Cue families on what they need to teach back to or demonstrate to their health care team.<br>This gives the health care providers an opportunity to confirm information and skills and correct misunderstandings.<br>-Help families feel confident that they can safely care for their child at home, know the signs of possible problems, know how to respond, and who to contact.	Education		
Phoenix Children's Hospital	Nuestro Camino y el autismo	Phoenix Children's	February 21, 2020	Spanish version of "Our Journey with Autism"	Education		
Phoenix Children's Hospital	Our Journey with a Kidney Transplant	Phoenix Children's	February 14, 2020	The Our Journey with Kidney Transplant app is designed to:<br>— facilitate conversations between families and the health care team.<br>— help parents identify what they need to know to safely care for their child at home.<br>— give parents a safe way to admit what they do not know or understand and ask questions.<br>— cue families on what they need to teach back or demonstrate to their nurse or doctor. This gives the health care provider an opportunity to confirm information and skills and correct misunderstandings.<br>— help families feel confident that they can safely care for their child at home after discharge from the hospital, know the signs of possible problems, and know how to respond.	Education		
Phoenix Children's Hospital	Nuestro Camino y el trasplante de riñón	Phoenix Children's	February 14, 2020	Spanish version of "Our Journey with a Kidney Transplant"	Education		
Phoenix Children's Hospital	Our Journey: Heart Transplant	Phoenix Children's	February 18, 2020	The Our JourneyTM with Heart Transplant app is designed to:<br>— Be used from the time of admission throughout hospitalization, to facilitate conversations between families and the health care team.<br>— Help parents identify what they need to know before going home.<br>— Give parents a safe way to admit what they don't know or understand, and ask questions.<br>— Cue families on what they need to teach back to or demonstrate for their nurse or doctor.<br>This gives the health care provider an opportunity to confirm information and skills and correct misunderstandings.<br>— Help families feel confident that they can safely care for their child at home after discharge from the hospital, know the signs of possible problems, and know how to respond.	Education		
Phoenix Children's Hospital	Nuestro Camino y el trasplante de corazón	Phoenix Children's	February 21, 2020	Spanish version of "Our Journey with a Heart Transplant"	Education		
Phoenix Children's Hospital	Our Journey with Heart Surgery	Phoenix Children's	February 17, 2020	The Our JourneyTM with Heart Surgery app is designed to:<br>— Be used from the time of admission throughout hospitalization, to facilitate conversations between families and the health care team.<br>— Help parents identify what they need to know before going home.<br>— Give parents a safe way to admit what they don't know or understand, and ask questions.<br>— Cue families on what they need to teach back to or demonstrate for their nurse or doctor.<br>This gives the health care provider an opportunity to confirm information and skills and correct misunderstandings.<br>— Help families feel confident that they can safely care for their child at home after discharge from the hospital, know the signs of possible problems, and know how to respond.	Education		
Phoenix Children's Hospital	Nuestro Camino y la cirugía del corazón	Phoenix Children's	February 21, 2020	Spanish version of "Our Journey with Heart Surgery"	Education		
Phoenix Children's Hospital	Our Journey: Doctor's Office	Phoenix Children's	February 18, 2020	The Our JourneyTM in the Doctor's Office is designed to:<br>— To facilitate conversations between families and the health care team.<br>— Help parents identify what they need to know before going home.<br>— Give parents a safe way to admit what they don't know or understand, and ask questions.<br>— Cue families on what they need to teach back to or demonstrate for their nurse or doctor.<br>This gives the health care provider an opportunity to confirm information and skills and correct misunderstandings.<br>— Help families feel confident that they can safely care for their child at home after leaving the doctor's office, know the signs of possible problems, and know how to respond.	Education		
Phoenix Children's Hospital	Nuestro Camino y el consultorio médico	Phoenix Children's	February 21, 2020	Spanish version of "Our Journey: Doctor's Office"	Education		

Phoenix Children's Hospital	Our Journey with Surgery	Phoenix Children's	February 18, 2020	The Our Journey™ with Surgery app is designed to:<br>— Be used from the time of admission throughout hospitalization, to facilitate conversations<br>between families and the health care team.<br>— Help parents identify what they need to know before going home.<br>— Give parents a safe way to admit what they don't know or understand, and ask questions.<br>— Cue families on what they need to teach back to or demonstrate for their nurse or doctor.<br>This gives the health care provider an opportunity to confirm information and skills and correct misunderstandings.<br>— Help families feel confident that they can safely care for their child at home after discharge<br>from the hospital, know the signs of possible problems, and know how to respond.	Education
Phoenix Children's Hospital	Nuestro Camino y la cirugía	Phoenix Children's	February 21, 2020	Spanish version of "Our Journey with Surgery"	Education
Phoenix Children's Hospital	Our Journey: Liver Transplant	Phoenix Children's	February 19, 2020	The Our Journey™ with Liver Transplant app is designed to:<br>— Be used from the time of admission throughout hospitalization, to facilitate conversations<br>between families and the health care team.<br>— Help parents identify what they need to know before going home.<br>— Give parents a safe way to admit what they don't know or understand, and ask questions.<br>— Cue families on what they need to teach back to or demonstrate for their nurse or doctor.<br>This gives the health care provider an opportunity to confirm information and skills and correct misunderstandings.<br>— Help families feel confident that they can safely care for their child at home after discharge<br>from the hospital, know the signs of possible problems, and know how to respond.	Education
Phoenix Children's Hospital	Nuestro Camino y el trasplante hepático	Phoenix Children's	February 21, 2020	Spanish version of "Our Journey with a Liver Transplant"	Education
Phoenix Children's Hospital	Our Journey in the ICU	Phoenix Children's	February 17, 2020	The Our JourneyTM in the NICU app is designed to:<br>— Be used from the time of admission throughout hospitalization, to facilitate conversations between families and the health care team.<br>— Help parents identify what they need to know before going home.<br>— Give parents a safe way to admit what they don't know or understand, and ask questions.<br>— Cue families on what they need to teach back to or demonstrate for their nurse or doctor.<br>This gives the health care provider an opportunity to confirm information and skills and correct misunderstandings.<br>— Help families feel confident that they can safely care for their child at home after discharge from the hospital, know the signs of possible problems, and know how to respond.	Education
Phoenix Children's Hospital	Nuestro Camino en NICU	Phoenix Children's	February 21, 2020	Spanish version of "Our Journey in the ICU"	Education
Phoenix Children's Hospital	Our Journey in the Hospital	Phoenix Children's	February 14, 2020	The Our JourneyTM in the Hospital is designed to:<br>— Be used from the time of admission throughout hospitalization, to facilitate conversations between families and the health care team.<br>— Help parents identify what they need to know before taking their child home safely.<br>— Give parents a safe way to admit what they don't know or understand, and ask questions.<br>— Cue families on what they need to teach back to or demonstrate for their nurse or doctor.<br>This gives the health care provider an opportunity to confirm information and skills and correct misunderstandings.<br>— Help families feel confident that they can safely care for their child at home after discharge from the hospital, know the signs of possible problems, and know how to respond.	Education
Phoenix Children's Hospital	Nuestro Camino en el hospital	Phoenix Children's	February 20, 2020	Spanish version of "Our Journey in the Hospital"	Education
Phoenix Children's Hospital	Our Journey with Tracheostomy<br>Phoenix Children's	Phoenix Children's	February 18, 2020	The Our JourneyTM with Tracheostomy app is designed to:<br>— Be used from the time of admission throughout hospitalization, to facilitate conversations between families and the health care team.<br>— Help parents identify what they need to know before going home.<br>— Give parents a safe way to admit what they don't know or understand, and ask questions.<br>— Cue families on what they need to teach back to or demonstrate for their nurse or doctor.<br>This gives the health care provider an opportunity to confirm information and skills and correct misunderstandings.<br>— Help families feel confident that they can safely care for their child at home after discharge from the hospital, know the signs of possible problems, and know how to respond.	Education
Phoenix Children's Hospital	Nuestro Camino y la traqueostomía	Phoenix Children's	February 20, 2020	Spanish version of "Our Journey with Tracheostomy"	Education
Phoenix Children's Hospital	Our Journey with Cancer	Phoenix Children's	February 20, 2020	The Our JourneyTM with Cancer app is designed to:<br>— facilitate conversations between families and the health care team.<br>— help parents identify what they need to know to safely care for their child at home.<br>— give parents a safe way to admit what they do not know or understand and ask questions.<br>— Cue families on what they need to teach back or demonstrate to their nurse or doctor.<br>This gives the health care provider an opportunity to confirm information and skills and<br>correct misunderstandings.<br>— help families feel confident that they can safely care for their child at home after discharge<br>from the hospital, know the signs of possible problems, and know how to respond.	Education
Phoenix Children's Hospital	Nuestro Camino y el cáncer	Phoenix Children's	February 20, 2020	Spanish version of "Our Journey with Cancer"	Education
Phoenix Children's Hospital	Our Journey with a Stem Cell Transplant	Phoenix Children's	February 20, 2022	The Our JourneyTM with Stem Cell Transplant app is designed to:<br>— facilitate conversations between families and the health care team.<br>— help parents identify what they need to know to safely care for their child at home.<br>— give parents a safe way to admit what they do not know or understand and ask questions.<br>— cue families on what they need to teach back or demonstrate to their nurse or doctor.<br>This gives the health care provider an opportunity to confirm information and skills and<br>correct misunderstandings.<br>— help families feel confident that they can safely care for their child at home after discharge<br>from the hospital, know the signs of possible problems, and know how to respond.	Education
Phoenix Children's Hospital	Nuestro Camino y el trasplante de células madre	Phoenix Children's	February 20, 2020	Spanish version of "Our Journey with Stem Cell Transplant"	Education
Phoenix Children's Hospital	Our Journey with Dialysis	Phoenix Children's	February 19, 2020	The Our Journey™ with Peritoneal Dialysis app is designed to:<br>— Be used from the time of admission throughout hospitalization, to facilitate conversations<br>between families and the health care team.<br>— Help parents identify what they need to know before going home.<br>— Give parents a safe way to admit what they don't know or understand, and ask questions.<br>— Cue families on what they need to teach back to or demonstrate for their nurse or doctor.<br>This gives the health care provider an opportunity to confirm information and skills and correct misunderstandings.<br>— Help families feel confident that they can safely care for their child at home after discharge<br>from the hospital, know the signs of possible problems, and know how to respond.	Education
Phoenix Children's Hospital	Nuestro Camino y la diálisis peritoneal	Phoenix Children's	February 20, 2020	Spanish version of "Our Journey with Dialysis"	Education
Phoenix Children's Hospital	Our Journey with Diabetes	Phoenix Children's	February 17, 2020	The Our JourneyTM with Diabetes app is designed to:<br>— Be used from the time of admission throughout hospitalization, to facilitate conversations between families and the health care team.<br>— Help parents identify what they need to know before going home.<br>— Give parents a safe way to admit what they don't know or understand, and ask questions.<br>— Cue families on what they need to teach back to or demonstrate for their nurse or doctor.<br>This gives the health care provider an opportunity to confirm information and skills and correct misunderstandings.<br>— Help families feel confident that they can safely care for their child at home after discharge from the hospital, know the signs of possible problems, and know how to respond.	Education
Phoenix Children's Hospital	Nuestro Camino y la diabetes	Phoenix Children's	February 20, 2020	Spanish version of "Our Journey with Diabetes"	Education
Phoenix Children's Hospital	Our Journey with Tube Feeding	Phoenix Children's	February 19, 2020	The Our Journey™ with Tube Feeding app is designed to:<br>— Be used from the time of admission throughout hospitalization, to facilitate conversations<br>between families and the health care team.<br>— Help parents identify what they need to know before going home.<br>— Give parents a safe way to admit what they don't know or understand, and ask questions.<br>— Cue families on what they need to teach back to or demonstrate for their nurse or doctor.<br>This gives the health care provider an opportunity to confirm information and skills and correct misunderstandings.<br>— Help families feel confident that they can safely care for their child at home after discharge<br>from the hospital, know the signs of possible problems, and know how to respond.	Education
Phoenix Children's Hospital	Nuestro Camino y la alimentación por sonda	Phoenix Children's	February 20, 2020	Spanish version of "Our Journey with a Feeding Tube"	Education
Phoenix Children's Hospital	Our Journey with a Central Line for Home Use	Phoenix Children's	January 31, 2020	The Our Journey with Central Line app is designed to:<br>— facilitate conversations between families and the health care team.<br>— help parents identify what they need to know to safely care for their child at home.<br>— give parents a safe way to admit what they do not know or understand and ask questions.<br>— cue families on what they need to teach back or demonstrate to their nurse or doctor. This gives the health care provider an opportunity to confirm information and skills and correct misunderstandings.<br>— help families feel confident that they can safely care for their child at home after discharge from the hospital, know the signs of possible problems, and know how to respond.	Education
Phoenix Children's Hospital	Nuestro Camino y el catéter central en la casa	Phoenix Children's	February 14, 2020	Spanish version of "A Journey with a Central Line"	Education
Phoenix Children's Hospital	Our Journey through High Risk Pregnancy	Phoenix Children's	February 14, 2020	The Our Journey through High Risk Pregnancy app is designed to:<br><br>— facilitate conversations between families and the health care team.<br>— help parents identify what they need to know to safely prepare for the birth of their baby.<br>— give parents a safe way to admit what they do not know or understand and ask questions.<br>— cue families on what they need to teach back or demonstrate to their nurse or doctor. This gives the health care provider an opportunity to confirm information and skills and correct misunderstandings.<br>— help families feel confident that they can safely prepare for the birth of their baby, know the signs of possible problems, and know how to respond.	Education
Phoenix Children's Hospital	Nuestro Camino y el embarazo de alto riesgo	Phoenix Children's	February 14, 2020	Spanish Version of "Our Journey through High Risk Pregnancy"	Education
Phoenix Children's Hospital	Our Journey with Ketogenic Diet Therapy	Phoenix Children's	February 17, 2020	The Our Journey with Ketogenic Diet Therapy app is designed to:<br><br>— facilitate conversations between families and the health care team.<br>— help parents identify what they need to know to safely care for their child at home.<br>— give parents a safe way to admit what they do not know or understand and ask questions.<br>— cue families on what they need to teach back or demonstrate to their nurse or doctor. This gives the health care provider an opportunity to confirm information and skillsand correct misunderstandings.<br>— help families feel confident that they can safely care for their child at home after discharge from the hospital, know the signs of possible problems, and know how to respond.	Education
Phoenix Children's Hospital	Nuestro Camino y la dieta cetogénica	Phoenix Children's	February 17, 2020	Spanish version of "Our Journey with Ketogenic Diet Therapy"	Education
Phoenix Children's Hospital	Our Journey with Inflammatory Bowel Disease (IBI	Phoenix Children's	February 17, 2020	The Our Journey with IBD app is designed to:<br><br>— facilitate conversations between families and the health care team.<br>— help parents identify what they need to know to safely care for their child at home.<br>— give parents a safe way to admit what they do not know or understand and ask questions.<br>— cue families on what they need to teach back or demonstrate to their nurse or doctor. This gives the health care provider an opportunity to confirm information and skills and correct misunderstandings.<br>— help families feel confident that they can safely care for their child at home, know the signs of possible problems, and know how to respond.	Education
Phoenix Children's Hospital	Enfermedad inflamatoria intestinal (IBD)	Phoenix Children's	February 17, 2020	Spanish Version of "Our Journey with IBD"	Education
Phoenix Children's Hospital	Our Journey with Bones	Phoenix Children's	February 19, 2020	The Our Journey™ with Bones app is designed to:<br>— Be used from the time of admission throughout hospitalization, to facilitate conversations between families and the health care team.<br>— Help parents identify what they need to know before going home.<br>— Give parents a safe way to admit what they don't know or understand, and ask questions.<br>— Cue families on what they need to teach back to or demonstrate for their nurse or doctor.<br>This gives the health care provider an opportunity to confirm information and skills and correct misunderstandings.<br>— Help families feel confident that they can safely care for their child at home after discharge from the hospital, know the signs of possible problems, and know how to respond.	Education
Phoenix Children's Hospital	Our Journey with Sickle Cell Disease	Phoenix Children's	February 20, 2020	The Our JourneyTM with Sickle Cell app is designed to:<br>— facilitate conversations between families and the health care team.<br>— help parents identify what they need to know to safely care for their child at home.<br>— give parents a safe way to admit what they do not know or understand and ask questions.<br>— cue families on what they need to teach back or demonstrate to their nurse or doctor.<br>This gives the health care provider an opportunity to confirm information and skills and<br>correct misunderstandings.<br>— help families feel confident that they can safely care for their child at home, know the signs of<br>possible problems, and know how to respond.	Education
